# Microbiological Values of Rainwater Harvested in Adelaide

**DOI:** 10.3390/pathogens7010021

**Published:** 2018-02-08

**Authors:** Chirhakarhula Emmanuel Chubaka, Harriet Whiley, John W. Edwards, Kirstin E. Ross

**Affiliations:** Environmental Health, College of Science and Engineering, Flinders University, GPO Box 2100, Adelaide 5001, South Australia; harriet.whiley@flinders.edu.au (H.W.); john.edwards@flinders.edu.au (J.W.E.); kirstin.ross@flinders.edu.au (K.E.R.)

**Keywords:** rainwater, *Escherichia coli* (*E. coli*), total coliforms, public health, gastroenteritis, filters, potable

## Abstract

In Australia, rainwater is an important source of water for many households. Unlike municipal water, rainwater is often consumed untreated. This study investigated the potential contamination of rainwater by microorganisms. Samples from 53 rainwater tanks across the Adelaide region were collected and tested using Colilert™ IDEXX Quanti-Tray*/2000. Twenty-eight out of the 53 tanks (53%) contained *Escherichia coli*. Samples collected from ten tanks contained *E. coli* at concentrations exceeding the limit of 150 MPN/100 mL for recreational water quality. A decline in *E. coli* was observed in samples collected after prolonged dry periods. Rainwater microbiological values depended on the harvesting environment conditions. A relationship was found between mounted TV antenna on rooftops and hanging canopies; and *E. coli* abundance. Conversely, there was no relationship between seasonality and *E. coli* or roof and tank structure materials and *E. coli*. In several tanks used for drinking water, samples collected prior to and after filtration showed that the filtration systems were not always successful at completely removing *E. coli*. These results differed from a study undertaken in the laboratory that found that a commercially available in-bench 0.45 µm filter cartridge successfully reduced *E. coli* in rainwater to 0 MPN/100 mL. After running a total of 265 L of rainwater which contained high levels of *E. coli* through the filter (half of the advertised filter lifespan), the filter cartridge became blocked, although *E. coli* remained undetected in filtered water. The difference between the laboratory study and field samples could be due to improper maintenance or installation of filters or recontamination of the faucet after filtration. The presence of *E. coli* in water that is currently used for drinking poses a potential health concern and indicates the potential for contamination with other waterborne pathogens.

## 1. Introduction

Worldwide, rainwater harvesting has the potential to supplement surface and groundwater resources in areas that have inadequate water supply [1]. In Australia, there has been an increase in harvesting rainwater to supplement municipal water. Until recently, the Australian Federal Government and many State Governments were offering financial incentives to householders to install rainwater harvesting systems [2]. The incentives were intended to make substantial savings on municipal water, to alleviate the problem linked with water restriction measures; and to mitigate drought conditions [3]. This resulted in 34% of Australian homes having rainwater tanks installed on their properties [4]. The proportion of households with tanks on their properties was higher in South Australia and in Adelaide compared with other Australian States and major cities. In that period, 76% of families in regional South Australia and 34% in Adelaide used rainwater as their source of drinking water [4].

In the community, there is a general belief that rainwater can be used for drinking with limited treatment [5], and that unlike municipal water which is believed to contain contaminants, rainwater is of higher quality as stated by rainwater users in South East of Queensland [6]. The South Australian Health Department (SA Health) advice is that if rainwater is collected from a roof is clear and has little taste, and is collected from a well maintained catchment system and tank, it is probably safe to drink [7]. Like SA Health advice, recommendations from the enHealth is that drinking rainwater from a well maintained roof catchment and tanks represents a relatively low risk of illness [8]. This guidance is supported by epidemiological evidence from a study of 1016 children aged 4–6 years from regional South Australia who drank rainwater which found no difference in gastroenteritis incidents compared with their peers who drank centralized municipal water [9]. In those that drank rainwater, the frequent exposure to pathogens might have enhanced their immune system, as the system can control the pathogens before they become harmful [10]. However, this epidemiological evidence does not take into consideration vulnerable populations, including immunocompromised and the elderly, who may be at greater risk [11]. Pathogenic organisms, including *Aeromonas*, *Campylobacter*, *Legionella*, *Salmonella*, *Giardia* and *E. coli*, have been found in rainwater harvested in many locations across Australia [12]. There have also been several reported outbreaks of salmonellosis, giardiasis and cryptosporidiosis that have been linked to contaminated rainwater [8,13,14,15].

This investigation had three objectives, firstly, to determine whether the microbial content of rainwater reported elsewhere was similar in South Australia, secondly, to assess the number of householders filtering their rainwater and whether this filtration is successful and thirdly, to test whether a commercially available filtration system removed microbial contamination to an acceptable level for potable water.

## 2. Results

### 2.1. Microbiological Values of Harvested Rainwater

Total coliforms and *E. coli* were regularly detected in rainwater samples. Table 1 presents all the tanks that were positive for total coliforms or *E. coli*, the primary use of the rainwater, the tank and roof material, the presence or absence of a water filter, and factors linked to rainwater harvesting immediate environment.

**Figure 1:**
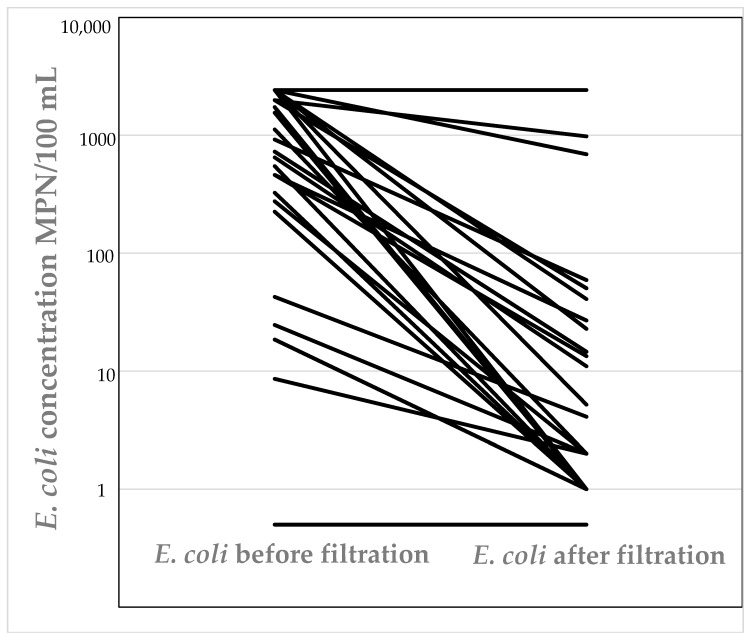
Concentration of *E. coli* present before and after filtration in rainwater tanks used for drinking water with filters fitted.

*E. coli* was detected in 53% (28 tanks) of all tanks that were surveyed (tanks were sampled 6–9 times over the course of this study—August 2015–August 2016). Of tanks that did not contain *E. coli*, 33% (18 tanks) contained total coliforms during all sampling rounds. Water collected from fifteen tanks exceeded the count of 200 MPN/100 mL for *E. coli*. Eleven out of the fifty-three tanks were plumbed-in, and the water used as source of drinking water. This represented 21% of tanks that were surveyed. Of these tanks, seven tanks (64%) were fitted with filtration systems for water sanitation (see Table 1). Samples collected from all seven tanks that had filtration systems installed contained *E. coli* and for more than one sampling event (Figure 1). The concentration of *E. coli* in samples collected post-filtration was overall statistically significantly less than in the pre-filtration samples (P ≤ 0.16 × 10 − 5); however, the filters did not always completely remove *E. coli*. Four tanks that were used for drinking that did not have filters fitted often contained *E. coli*.

Water from the remaining forty-two tanks (79% of tanks surveyed) were either used for indoor purposes such as toilet flushing or for outdoor purposes, such as gardening or firefighting. It should be noted that of the twenty-eight tanks that contained *E. coli*, ten tanks (19%) were plumbed-in for toilets flushing, and water from five tanks (9%) was used outdoor for gardening. In addition, water from one tank (2%) was used for firefighting and water from another tank (2% of tanks surveyed) was not used for any purpose.

The use of coliforms as indicator organisms has been previously debated; however, this study demonstrated that there was a relationship between the total number of coliforms (MPN/100 mL) and total number of *E. coli* (MPN/100 mL). This relationship is not a surprise given that *E. coli* is a coliform. The problem with using coliforms as indicator organisms is that they are not specifically of faecal origin and are present naturally in the environment. The linear regression (R^2^ = 0.145, and *p*-value < 0.01), indicates they are a reasonable estimate of fecal contamination The study observed no seasonality in bacteria load in samples however; a moderate relationship was observed between total coliforms and *E. coli* in samples collected during both winter and summer months. However, a difference in bacteria load was observed after significant period of no rainfall. Moreover, there was a relationship between rainwater temperature and *E. coli* abundance (R^2^ = 0.321, *p*-value < 0.001). Temperature values ranged from 5.7 °C to 29.5 °C. Thus, increased rainwater temperature was associated with increased *E. coli* concentrations. Conversely, the regression analysis found no linear relationship between rainwater pH and *E. coli* abundance (R^2^ = −0.005, and *p*-value = 0.17). Rainwater pH ranged from 4.5 to 8.8. Thirteen samples had a pH lower than 6.0, which is a level below which is potentially corrosive to structure materials, if the level of alkalinity is low [16].

### 2.2. Physical Environment around Rainwater Harvesting Systems

The building materials used in the catchment areas; building rooftops, gutters and tanks downpipes, were assessed to determine whether the building materials played a role in rainwater microbial contamination. It was found that there was no relationship between building structure materials and rainwater microbial content. In addition, hanging canopies and mounted TV antennas on building rooftops were identified. Having a TV antenna impacted on bacteria abundance in rainwater samples. It was found that 19% of samples that were harvested from building with mounted TV antennas contained *E. coli*. Likewise, 12% of samples harvested from catchments partially covered by hanging canopies contained *E. coli*. The regression analysis (R^2^ = 0.025, *p*-value < 0.001) indicated a weak but positive correlation between the presence of mounted TV antennas on building rooftops and hanging canopies, and *E. coli* abundance. Nearly 75% of catchment areas that were partially covered by trees canopies had mounted TV antennas installed. For the fifty-three tanks surveyed, there were no maintenance works reported on catchments areas, gutters, downpipes or tanks. No relationship between having a first flush device and *E. coli* and total coliform content was found (R^2^ = 0.003, *p*-value = 0.956 and R^2^ = 0.003, *p*-value = 0.53 respectively).

### 2.3. Investigating the Efficacy of a Water Filtration System to Remove Microbial Contamination

A laboratory study was conducted to evaluate the efficacy of a commercially available filter to remove microorganisms. It was found that before filtration, 93% of samples contained total coliforms and 54% contained *E. coli*. The organism count ranged from <1.0 MPN/100 mL–≥2419.6 MPN/100 mL for both total coliforms and *E. coli*. Filtration reduced the organisms count to 0 MPN/100 mL in all samples for target bacteria. This indicated that under laboratory conditions the filtration system is able to remove bacteria from rainwater. The filter reduced rainwater bacterial load to acceptable safety limit of 0 MPN/100 mL for *E. coli* in drinking water.

The bench mounted filtration unit reached clogging point was reached after 265 L rainwater had passed through the filter over a 6 months period (half of Puratap^®^ cartridge’s advertised lifespan). Since a little water was left in the two cartridges (1 L/cartridge) at the filter clogging point, this water was tested for bacteria. The intention was to verify whether the bacteria could still grow and be detected in the water that remained in the cartridges while undetected in filtered water. In the inlet cartridge (membrane cartridge), organisms count for total coliforms was ≥2419.6 MPN/100 mL, and 55.4 MPN/100 mL for *E. coli*. Likewise, in the outlet cartridge (activated carbon cartridge), organisms count was 178.9 MPN/100 mL for total coliforms and 48.7 MPN/100 mL of *E. coli*. This suggests that the bacteria could still be detected in the water in both cartridges while its passage through the filtration unit remained blocked, and no bacteria could be detected in rainwater collected at the top end of the connected tap.

## 3. Discussion

In Australia, *E. coli* guidelines for drinking water is ≤0 MPN/100 mL [17]. Nearly 54% (197/365) of samples tested in this study were found to be positive for *E. coli*. Of this total, 60 samples (30%) that contained *E. coli* were collected from 11 tanks. This represented 21% of tanks surveyed. These tanks were plumbed-in and fitted with filters, and the water used as source of drinking water. These findings are higher compared with the study conducted in 2007 in regional South Australia that found 30% of 974 rainwater samples collected from 325 rainwater tanks were positive for *E. coli* [16].

The most significant findings from this study was the number of tanks that had filters fitted and still tested positive to *E. coli*. This is consistent with White, et al. [18] who suggests that not all filters are designed to remove bacteria from rainwater. Thus, it was found that all water samples from tanks that were fitted with filtration systems contained *E. coli*, on more than one occasion (Table 1). These results contradicted the findings from the experimental, laboratory testing of a Puratap^®^ filter. Laboratory testing demonstrated that a Puratap^®^ filter effectively removed all *E. coli* and total coliform contamination from the rainwater. Notably, the filter only managed 1/10 of its advertised filtration capacity before becoming blocked. In tanks with higher suspended solids, cartridge lifetime would even be shorter than the suggested period. The experimental study found that at the unit clogging point, the filter capacity to retain bacteria remained effective and no bacteria was detected in filtered water at the top end of Puratap^®^ supplied outlet tap.

The difference in findings from field samples and the experimental study could be due to differences in flow rates and water pressures in the rainwater used in households compared to the experimental design. Alternatively, it could be indicative of inadequate maintenance of filters, which was supported by discussion with participants, many of whom indicated that the filter cartridges had never been replaced. Only one tank out of fifty three tanks was reported to have been drained and the bottom sludge removed. Contamination could occur with cartridges that have reached the end of their factory lifetime, or in cartridge with factory faults. It should be noted that not all filters meet the standard for bacteria removal from water. Otherwise, filters may have been originally improperly fitted or the contamination could have been from the faucet, post filtration.

In some marginal cases, external factors to the tanks could have contributed to the presence of *E. coli* in filtered water. Studies indicate that strains of *E. coli* can survive and even grow in an open environment, subject to the environmental level of nutrients, and conditions such as temperature and pH [19]. Bacteria could be associated with rainwater droplets during rainfall events and be in connecting pipes, or water exposure to ambient air could facilitate the incursion of these organisms into filtered water [20]. After that, the bacteria can grow inside pipes or in the faucet post filter and ultimately, be detected in filtered water at the point of collection. On the other hand, filter cartridges pore size could have been larger than *E. coli* size and allow the bacteria pass through the system unblocked and remain in the filtered water.

It should be noted that *E. coli* size vary from 0.5 μm in width and 2 μm in length [21]. Thus, filters with cartridge pore size smaller than *E. coli* size would remove the bacteria from water, providing the filters are regularly maintained, and the cartridge replaced after the suggested factory lifetime. However, case studies have indicated that membrane cartridges of 0.2 µm−0.22 µm are benchmarks for bacteria retention from water [21,22]. Well maintained, these cartridges can be effective in removing *E. coli*, Salmonella (2μm by 0.5 μm), Campylobacter (0.2−0.8 × 0.5−5 µm), Enterococci (0.6–2.0 μm by 0.6–2.5 μm), Giardia (10–15 µm), Legionella (2 µm by 0.3–0.9 µm) and Aeromonas (0.3 to 1.0 μm by 1.0 to 3.0 μm) from rainwater, but challenges remain on these cartridges capacity to remove viruses that may occur in rainwater. Viruses vary in size from 27 nm to 250 nm diameter, and a nanometer (nm) corresponds to one-thousandth of a micrometer (µm) [23]. Filters with cartridges of 0.45 µm pore size were accepted by Lee and Deininger [24] as benchmark for bacteria retention. It should be noted that in below ground tanks, sewerage effluent can be discharged by surface runoff into poorly sealed tanks, and tanks that have cracks can allow human infectious protozoa and viruses into stored rainwater [25]. The likelihood of finding these organisms in rainwater collected from above ground tanks is low [26]. In order to avoid virus contamination, a membrane filtration of 0.01 µm–0.1 µm and ultraviolet disinfection can be use [27]. 

However, the cost to acquire and to maintain these highly efficient systems is high, and their small pore sizes can trigger an early blockage when applied on rainwater with high sediments [28]. Such filters were not tested in this study. It was found that 100% of tanks (seven tanks) that had filters fitted tested positive to *E. coli* at least once, suggesting issues of filter maintenance and cartridge replacement. Only one tank (2% of tanks surveyed) was reported to have been drained for bottom sludge removal.

In Australia, incidents of illness linked with drinking rainwater are low even though rainwater collected in many areas fails to meet the Australian Drinking Water Guideline microbiological standard requirements [8]. Similar findings on rainwater quality were reported by Ahmed, Gardner and Toze [26] and Ahmed, et al. [29] who suggested that members of the public avoid drinking untreated rainwater, particularly older and immunocompromised people. Notably, many samples collected in the Adelaide region were found to contain *E. coli* above the guideline levels for recreational water, suggesting that rainwater was not even fit for recreational use. In Australia, organism count should not exceed the threshold of 150 fecal coliforms/100 mL in recreational water for five consecutive sampling events, and sampling should be at regular intervals and extended to a period of 30 days [30] 

The guideline for recreational water for *E. coli* is set to a more stringent limit of 126 organisms/100 mL in New Zealand. Although links existed between *E. coli* and rainwater fecal contamination, no study in bacteria speciation has been carried out to whether determine whether *E. coli* found in rainwater is Enterohemorrhagic *E. coli* O157:H7. Further research could be undertaken to assess whether it is a possibility. However, for domestic above ground tanks, the risk of detecting *E. coli* O157:H7 strain in harvested rainwater would be negligible, and associated health risks low in magnitude.

When considering the observed *E. coli* prevalence, rainwater harvested in the Adelaide region may pose a risk when used for toilet flushing or gardening without a minimum level of disinfection. During gardening or toilet flushing, incidents of contamination could potentially occur through inhalation of droplets and aerosols that contain *E. coli* or other pathogenic microorganisms. However, risks of infection through these routes are lower than those encountered through drinking [31]. Additionally, rainwater with high *E. coli* content would not be recommended for watering fruit and vegetable plants as bacteria can colonise the roots and the leaf and on harvest spread in the food processing chain and cross contaminate other food products [19]. In the Adelaide region, it was found that many tanks exceeded the maximum detection limit of 200 CFU/100 mL for *E. coli*, for water intended for irrigation [32]. A less stringent limit of 250 CFU/100 mL for plants watering exists in the United Kingdom (BS8515:2008) [33]. In Australia, water must not exceed the threshold of 10 CFU/100 mL for raw human food crop watering, 100 CFU/100 mL for grazing animal other than pigs and dairy animals, and <1000 CFU/100 mL for grazing dairy animals with a withholding period of five days [34]. Alternatively, such water should not be recommended for playgrounds and school yards watering, if attended by small children as they have a high incidence of hand-to-mouth action. Freshly watered playground and school yards have higher contamination potential as bacteria can survive longer on grassy surfaces with higher moisture conditions [35]. This study found a decline in the number of bacteria in samples collected after a prolonged drier period. This could be due to higher temperatures causing deposited fecal matter on structures to dry out more quickly and kill the bacteria. It should be noted that in this study, rainwater samples were collected from galvanised and tiled catchments, subject to a range of humidity parameters, and to changing ambient temperatures. Outside the host vector, *E. coli* lifespan can be compromised on dry surfaces where humidity is low. Experiments have shown a reduction in *E. coli* up to 99.9%, following 24 h direct exposure to light [36]. Moreover, *E. coli* survival may be compromised on dry surfaces after 120 min at 20 °C and 360 min at 4 °C on metallic surfaces [37].

To some degree, the poor record of tank maintenance might be linked with the design of the tank. All tanks surveyed had large inlets in the rainwater intake region, and overflow valves; but no tank had sludge valve for tank drainage; and tanks’ outlets were limited to taps used for rainwater collection. It is suggested that the next generation of tanks have larger outlet valves in the sludge zone to allow easy tank sludge removal. This can assist households to clean their tanks and avoid the costs associated with having the water tank professionally cleaned.

## 4. Materials and Methods 

### 4.1. Study Area and Samples Collection

**Figure 2:**
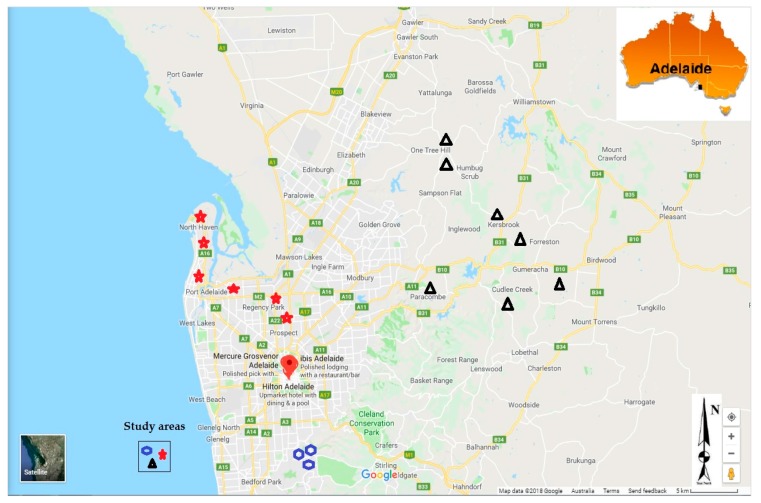
Study area showing the location of rainwater tanks sampled (2018, Google Maps).

The samples were collected from July 2015 to August 2016 in the Adelaide region (Figure 2). The study was approved by Flinders University Social and Behavioural Research Ethics Committee (SBREC N° 6782, and 6782 SBREC modification N° 2) in compliance with the National Statement on Ethical Conduct in Human Research (NSECHR). Rainwater tanks were randomly identified across the Adelaide region. Participants were asked a few questions about their water tanks and water usage, and whether maintenance works were carried on catchments areas and on tanks. This included bottom tank sludge drainage, gutters and downpipes to tank cleaning, tank age, whether they had first flush devices installed, whether they had filters installed and regularly maintained, whether the tanks were plumbed directly into the house and what the water was primarily used for.

Additional details on tanks and roofs structure materials were made via observation. The rainwater samples were collected in 1 L rinsed, acid-washed polyurethane bottles. During sample collection, the water was run for several seconds before collecting. For tanks that had water filtration systems fitted and were plumbed-in and the water used for drinking, homeowners were asked to provide a sample of water from an indoor tap for analysis. For those tanks, an unfiltered sample was also directly collected from the tank. Freshly collected samples were transported back to the laboratory in an esky on ice and processed immediately on arrival. Samples were tested for total coliforms, and for *E. coli* in a time not exceeding 24 h after collection.

Water parameters such as water pH and water temperature were taken in the field, at the time of sampling. A digital PH-618 Pen-Type Automatic Calibration IP65 Waterproof PH Meter was used for rainwater pH and temperature recording (Shenzhen Handsome Technology Co., Ltd., Guangdong, China; Walcom Int’l Industry Ltd., Hong Kong, China). 

A total of 365 rainwater nsamples were collected in the Adelaide region from 53 different tanks, with 120 samples collected in the Adelaide plains from 18 tanks, 97 samples in the Adelaide foothills from 15 tanks, and 148 samples in the Adelaide Hills from 20 tanks. Samples were collected every month or after storm events that occurred between two scheduled sampling dates, for a period of one year. Many tanks did not have water for sampling in summer, following the drier conditions that prevailed in the region in summer months.

### 4.2. Samples Processing and Testing

Total coliforms and *E. coli* was enumerated with the Colilert™ IDEXX Quanti-Tray*/2000 water testing method using the standard procedure (IDEXX Laboratories, Inc., Westbrook, ME, USA). Briefly, a Collilert*-18 reagent was added to undiluted and unfiltered rainwater samples in a 100 mL sterile polyurethane container. Then the sample was transferred in a Quanti-Tray*/2000, a semi-automated total coliform and *E. coli* enumeration method based on the Most Probable Number (MPN) model. The Quanti-Tray*/2000 was sealed in a Quanti-Tray*/2000 Sealer, Model 2X (IDEXX Laboratories, Inc., Westbrook, ME, USA). After sealing, the Quanti-Tray*/2000 was immediately incubated for 24 h at 37 °C. At the end of the incubation time, coliform positive reaction appeared in yellow wells, and *E. coli* positive fluoresces under 6-watt, 365 nm long-wave ultra violet lamp. Organism numbers was estimated by means of the Most Probable Number (MPN).

### 4.3. Investigating Efficacy of Water Filter to Remove Microbial Contamination

The effectiveness of a commercially available filter to remove microbial contamination was assessed. The investigation was based on Puratap^®^ Pty Ltd. filter performance claims. The Puratap^®^ Ultrafiltration Filter advertising material states that the filter “protects rainwater consumers against faecal coliforms, bacteria and viruses” [38]. Similarly, the Amway eSpring™ Water Filter states that it effectively destroys over 99% of bacteria (*E. coli*, *Aeromonas hydrophila*, *Campylobacter jujeni*, *Salmonella*, *Legionella pneumophila*, *Klebsiella terrigena*, *Vibrio cholera*, *Yesinia entertocolitica* and *Shigella dysenteriae*), viruses and protozoan parasites from rainwater [39]. Fifty-three ×5 L rainwater samples were collected from January to June 2016 (from one tank known to have high levels of *E. coli* contamination) and run through the filter system. Each sample of 5 L of unfiltered rainwater was run through a Puratap^®^ double cartridge filter mounted with MasterFlex tubing connectors, consisting of a pre-sediment cartridge (membrane cartridge) of 1 µm/pore size, and an activated carbon cartridge of 0.45 µm/pore size, using a powered Cole-Parmer MasterFlex Peristaltic^®^ L/S pump, Model 7553-79 and a Cole-Parmer MasterFlex L/S Modular Controller 7553-78 (Cole-Parmer 625-Vernon Hills, IL 60061, United States). The filtered sample was collected from a Puratap^®^ supplied outlet tap top-end, in a 100-mL sterile polyurethane container. Duplicate samples of both unfiltered and filtered were tested for *E. coli* and total coliforms using the Colilert™ IDEXX Quanti-Tray*/2000 water.

### 4.4. Statistical Analysis

Data in the study were graphed using Microsoft Excel (Microsoft Corporation, Washington, WC, USA), and analysed using IBM SPSS statistical software package (IBM SPSS Statistics for Windows, Version 23.0. IBM Corp, Armonk, NY, USA), and GraphPad Prism 7.02 (GraphPad Software, Inc., San Diego, CA, USA). The bivariate correlation by means of Test between Subjects-Effects (3 Way-ANOVA) was used to measure the correlation and linear regression between variables. Data statistical significance was set to the statistical value of *p*-value < 0.05 against the null hypothesis. A paired *t*-test was used to determine influence of filtration on the bacterial load (*p*-value < 0.05).

## 5. Conclusions

This study demonstrates that rainwater harvested in the Adelaide region is of poor microbiological quality for drinking. Out of a total of fifty-three tanks surveyed, twenty-eight tanks (53%) were tested positive at least once for total coliforms and *E. coli* and 10 tanks contained *E. coli* at concentrations that exceeded the accepted threshold of 150 CFU/100 mL for recreational water. This is a significant public health concern as this water is being used by many Adelaide households as source of drinking water. In tanks with filters fitted, the concentration of E.coli was consistently reduced, but not completely removed. These results contradicted laboratory testing of a bench mounted filtration unit that successful reduce total coliforms and *E. coli* to 0/100 MPN.

The study found little evidence linking rainwater microbiological quality and structure materials however; links were observed between rooftops mounted TV antennas and rainwater bacteriological quality. Most of the samples collected from tanks connected to rooftops with mounted TV antenna contained *E. coli*. Further research is needed to investigate the presence of other pathogenic microorganism and their potential to be removed by filters. A greater exploration of the effectiveness of filters and reasons for their failure is also needed.

## Figures and Tables

**Table 1:** Number of samples found positive to total coliforms and *E. coli* in some tank.

Tank Number	Rainwater Use	Filter on Tank	Positive to Total Coliforms/8 Samples	Positive to *E. coli*/8 Samples	Total Coliforms: Minimum and Maximum Detection	*E. coli*: Minimum and Maximum Detection	Roof Material	Tank Material	TV Antenna on Rooftop	Hanging Canopy
1	Drinking	No	8	6	18.5–2419.6	1.0–59.1	Galvanized	Galvanized	Yes	No
2	Drinking	Yes (before filter)	8	6	1046.2–613.1	866.4–461.1	Galvanized	Polyethylene	Yes	No
		Yes (after filter)	8	5	28.8–365.4	1.0–13.4				
3	Drinking	Yes (before filter)	7	4	58.6–≥2419.6	11.0–365.4	Galvanized	Polyethylene	Yes	No
		Yes (after filter)	5	2	42.6–866.4	1.0–5.2				
4	Drinking	No	8	5	1.0–≥2419.6 *	1.0–980.4	Galvanized	Polyethylene	Yes	No
5	Drinking	Yes (before filter)	8	7	140.8–≥2419.6 *	13.2–1986.3	Tiles	Polyethylene	No	Yes
		Yes (after filter)	7	5	172.3–648.8	2.0–50.4				
6	Drinking	No	8	6	5.2–≥2419.6 *	1.0–7.2	Tiles	Polyethylene	Yes	Yes
7	Drinking	Yes (before filter)	8	5	1.0–≥2419.6 *	1.0–≥2419.6 *	Galvanized	Galvanized	No	Yes
		Yes (after filter)	6	3	1.0–≥2419.6 *	1.0–≥2419.6 *				
8	Drinking	No	6	3	2.0–≥2419.6 *	1.0–39.3	Galvanized	Concrete	No	No
9	Drinking	Yes (before filter)	8	3	256.5–≥2419.6 *	2.0–22.8	Galvanized	Polyethylene	Yes	No
		Yes (after filter)	2	1	24.3–1553.1	<1.0–1.0				
10	Drinking	Yes (before filter)	6	3	32.7–1986.3	13.4–648.8	Galvanized	Polyethylene	Yes	Yes
		Yes (after filter)	3	2	3.0–88.2	1.0–11.0				
11	Drinking	Yes (before filter)	6	3	29.9–≥2419.6 *	1.0–24.6	Galvanized	Polyethylene	Yes	Yes
		Yes (after filter)	4	1	8.6–≥2419.6 *	<1.0–2.0				
12	Gardening	No	8	6	151.5–≥2419.6 *	3.2–111.2	Tiles	Galvanized	Yes	Yes
13	Gardening	No	8	7	83.6–≥2419.6 *	6.0–648.8	Tiles	Polyethylene	No	Yes
14	Gardening	No	6	3	4.1–1986.3	4.1–38.9	Tiles	Galvanized	Yes	No
15	Gardening	No	6	5	24.6–1203.3	1.0–33.1	Tiles	Galvanized	Yes	No
16	Gardening	No	6	4	18.5–1119.9	1.0–517.2	Tiles	Galvanized	Yes	No
17	Toilets flushing	No	8	2	22.9–307.6	1.0–2.0	Galvanized	Galvanized	No	No
18	Toilets flushing	No	8	8	14.4–259.5	1.0–27.5	Galvanized	Polyethylene	No	No
19	Toilets flushing	No	8	6	179.3–1413.6	2.0–461.1	Tiles	Polyethylene	No	No
20	Toilets flushing	No	8	6	142.1–1299.9	3.1–1203.3	Tiles	Polyethylene	No	No
21	Toilets flushing	No	8	6	165.0–≥2419.6 *	1.0–1986.3	Tiles	Polyethylene	No	No
22	Toilets flushing	No	6	2	17.5–107.6	9.7–14.8	Tiles	Polyethylene	Yes	No
23	Toilets flushing	Yes	8	6	18.9–≥2419.6 *	2.0–435.2	Galvanized	Polyethylene	No	No
24	Toilets flushing	No	5	1	16.4–≥2419.6 *	1.0–107.1	Galvanized	Polyethylene	No	Yes
25	Toilets flushing	No	6	4	119.9–≥2419.6 *	2.0–18.7	Galvanized	Galvanized	No	No
26	Toilets flushing	No	8	8	27.5–≥2419.6 *	1.0–≥2419.6 *	Galvanized	Polyethylene	No	No
27	Firefighting	No	6	1	5.2–1986.3	1.0–325.5	Galvanized	Polyethylene	Yes	No
28	Unused	No	6	6	13.5–1299.7	1.0–58.6	Tiles	Galvanized	Yes	Yes

* Count that exceeded the quantitation limit of 2416.9 MPN/100 mL in the IDEXX Quanti-Tray*/2000 Most Probable Number Table. * Not inclusive of samples that contained < 1.0 MPN, and samples only positive to total coliforms.
